# Body Dissatisfaction as a Mediator between Identity Formation and Eating Disorder Symptomatology in Adolescents and Emerging Adults

**DOI:** 10.5334/pb.564

**Published:** 2020-09-29

**Authors:** Nina Palmeroni, Koen Luyckx, Margaux Verschueren, Laurence Claes

**Affiliations:** 1 KU Leuven, BE; 2 University of Antwerp, BE; 3 University of the Free State, ZA

**Keywords:** identity formation, body dissatisfaction, eating disorder symptomatology, mid-to-late adolescence, emerging adulthood

## Abstract

**Objective:**

Eating disorder symptomatology generally develops during adolescence and emerging adulthood. Previous research has focused on the role of identity formation or body image in the development of eating disorder symptomatology, but integrative work is lacking. For this reason, the present cross-sectional study examined the mediating role of body dissatisfaction in the relation between identity formation and eating disorder symptomatology.

**Method:**

The sample comprised 659 participants between 15 and 30 years old (68.9% females; M_age_ = 19.44; SD_age_ = 3.99). All participants completed self-report measures on identity (*Self-concept and Identity Measure)*, body dissatisfaction *(the Body Image Feelings and Attitudes subscale of the Body Investment Scale and the Body Dissatisfaction subscale of the Eating Disorder Inventory-3)*, and eating disorder symptomatology *(Eating Disorder Inventory-3)*. Latent variable modeling from a structural equation modeling approach was used.

**Results:**

First, identity formation significantly predicted eating disorder symptomatology. Additionally, indirect effects were found linking identity formation to eating disorder symptomatology through the mechanism of body dissatisfaction. No significant differences between males and females or between adolescents and emerging adults on direct or indirect effects were found.

**Conclusion:**

The present study indicated that body dissatisfaction mediated the relationship between identity formation and eating disorder symptomatology during mid-to-late adolescence and emerging adulthood. Provided that the present findings can be replicated in a future longitudinal study, they demonstrate that both identity formation and body dissatisfaction should be taken into account in prevention and intervention programs targeting eating disorder symptomatology.

Adolescence and emerging adulthood are challenging periods. From puberty onwards, the body of adolescents undergoes rapid changes (Stice & Whitenton, 2002). The body of female adolescents generally changes in shape and size, and body fat increases (Wertheim & Paxton, 2011), whereas male adolescents generally grow taller, gain muscles, and their shoulder width increases (Ricciardelli & McCabe, 2011). Hence, male adolescents tend to move closer to male body ideals, whereas female adolescents experience specific bodily changes that bring them further away from body ideals imposed by media, parents, and/or peers. These body ideals consist of an unrealistically thin body for girls and a lean, muscular body for boys (Cash & Smolak, 2011). At the same time, opinions of others about their own appearance are highly significant, resulting in a strong focus on body and appearance in young people (Arnett, 2000).

This heightened focus on appearance almost invariably leads to discrepancies between actual and ideal body image, as body ideals involve levels of thinness and muscularity that are unattainable for most (Dittmar, 2007). As a result, many youth experience body dissatisfaction. Body dissatisfaction refers to the evaluative dimension of body image in which an individual experiences negative, dysfunctional feelings and beliefs towards one’s own body (e.g., weight and body shape) (Cash, Deagle, & Mikulka, 1997; Cash & Smolak, 2011). Individuals who experience body dissatisfaction wish to have body characteristics that are different from how they perceive their body, resulting in negative affect (Cash & Smolak, 2011). Body dissatisfaction in adolescents and emerging adults (and especially in females) is so highly prevalent that it is thought to be the norm rather than the exception (Rodin, Silberstein, & Striegel-Moore, 1984; Shagar, Harris, Boddy, & Donovan, 2017).

Considerable research attention has been devoted to the role of body dissatisfaction in the onset and course of eating disorder (ED) symptomatology (Brausch & Muehlenkamp, 2014; Stice, 2002). ED symptomatology generally develops and increases during late adolescence, often persisting or developing into a clinical ED in emerging adulthood (Slane, Klump, McGue, & Iacono, 2014; Stice, 1994, 2002). Although the role of body dissatisfaction has been widely addressed in ED symptomatology, specific key mechanisms that influence body dissatisfaction in adolescents and emerging adults have not been dealt with in depth. Identity formation, a key developmental task in these life periods, has been linked to body dissatisfaction and ED symptomatology (Nelson, Kling, Wängqvist, Frisén, & Syed, 2018; Vartanian, Hayward, Smyth, Paxton, & Touyz, 2018; Verschueren, Claes, et al., 2018; Verstuyf, Van Petegem, Vansteenkiste, Soenens, & Boone, 2014; Wängqvist & Frisén, 2013). However, no research to date has examined how these factors are interrelated and whether body dissatisfaction may mediate the relationship between identity formation and ED symptomatology. In this paper we examined this mediation model in a sample of mid-to-late adolescents and emerging adults.

## Identity Formation Throughout Adolescence and Emerging Adulthood

Identity formation constitutes a central developmental task during adolescence and emerging adulthood (Arnett, 2000). According to Erikson (1968), one’s identity structure can be defined by a feeling of sameness and continuity across time and contexts. Youth need to resolve an identity crisis triggering questions such as *‘Who am I?’,‘What do I want in life?’* (Erikson, 1968). Confronted with these questions, feelings of *identity confusion* can emerge, in which a clear sense of purpose is lacking. As adolescents grow older, their identity is expected to strengthen progressively. Individuals develop a sense of self-continuity in which different self-identified values and goals fit together in an integrated whole, described as *identity synthesis* (Erikson, 1968).

Due to the growing complexity of Western societies, the identity formation task is prolonged into the late teens and 20s (Arnett, 2000). Education is lengthened and important choices (e.g., parenthood) are delayed in time (Arnett, 2000), providing emerging adults with ample opportunities to explore before long-term commitments are made. However, many young people are uncertain where their explorations will lead them, resulting in insecurity and indecisiveness (Arnett, 2000). Accordingly, identity distress (i.e., feelings of distress over one or more identity choices) increases during emerging adulthood (Palmeroni et al., 2019).

A certain amount of identity confusion and distress is normative. Most young individuals follow a healthy identity formation trajectory in which they move from identity confusion into the direction of identity synthesis (Erikson, 1950, 1968; Meeus, 2011). Researchers indicated that, although many young people experience temporary identity concerns, for some people these concerns can lead to pathological identity disturbance (Erikson, 1950). According to developmental psychopathology, this kind of pathological identity disturbance exceeds normative identity confusion (Kaufman, Montgomery, & Crowell, 2014). Hence, identity functioning can be placed on a continuum ranging from healthy identity functioning to clinical identity disturbance (Kaufman et al., 2014). In this study, three aspects of identity will be considered: identity consolidation, identity disturbance, and lack of identity (Kaufman, Cundiff, & Crowell, 2015). Identity consolidation refers to healthy identity functioning, whereas identity disturbance refers to normative identity confusion and lack of identity represents clinical identity disturbance.

## Identity Formation as a Predictor of ED Symptomatology

Self- and identity-related problems have been forwarded in etiological models as important contributing factors to ED symptomatology. Casper (1983) mentioned that *‘the lack of a stable self-concept and secure self-regard predisposes adolescents to use thinness in a misguided strife for individuation’* (p. 388). Schupark-Neuberg and Nemeroff (1993) stated that patients suffering from bulimia nervosa generally lack a clear self, with ED symptomatology (e.g., binge eating) representing avoidance strategies to deal with distressing identity-related issues (Wheeler, Adams, & Keating, 2001). More recent studies (Verschueren, Claes, et al., 2018; Verschueren et al., 2017; Verstuyf et al., 2014) highlighted a significant relationship between identity formation and ED symptomatology. Verschueren and colleagues (2017) reported that patients with an ED experienced more identity-related issues than healthy controls. Verschueren and colleagues (2018) extended this finding in community adolescents and found that identity confusion positively predicted bulimia, whereas identity synthesis buffered against bulimia and drive for thinness. Bulimia, in turn, predicted an increase in identity confusion and a decrease in identity synthesis over time. Finally, individuals who avoid dealing with identity issues have been found to report less health-focused eating regulation (Verstuyf et al., 2014).

## Body Dissatisfaction as a Mediator between Identity Formation and ED Symptomatology

Although research has increasingly linked identity formation to ED symptomatology, little is known about intervening mechanisms. The present study investigated the possible mediating role of body dissatisfaction.

### Identity formation as a predictor of body dissatisfaction

In recent years, there has been a growing interest in the relationship between identity formation and body image (Daniels & Gillen, 2015). Erikson (1968) already conceptualized the body as the home to the self. Physical changes and sexual maturation during adolescence and emerging adulthood also spark questions about one’s identity (Erikson, 1968). During these life periods, body and appearance are highly significant, resulting in a strong focus on the body as a central identity aspect (Arnett, 2000; Harter, 1999), affecting the way youth perceive their identity in relation to their body (Frisén & Holmqvist, 2010; Nelson et al., 2018).

The importance of the body in identity formation is also highlighted in embodiment theorizing (Piran, 2016). For instance, an important dimension in the embodied experiences of females is the (dis)connection between body and self (Piran, 2016). Individuals that experience a body-self connection feel comfortable in their body and feel at home in their own body. However, individuals who experience a body-self disconnection feel uncomfortable in their body and experience the body as separate from the self. This results in negative feelings toward the body and a desire to control or change their body. In sum, people may convey their identity through their body, and how the body is experienced may impact identity formation.

Despite this theoretical interest, only a few studies directly examined the link between identity formation and body image. Late adolescents’ identity exploration and commitment have been linked to body-esteem and body ideal internalization (Wängqvist & Frisén, 2013). Kamps and Berman (2011) demonstrated that negative body image and identity distress are related. Similarly, adolescents with decreasing weight/appearance-esteem over time experienced lower identity coherence during emerging adulthood (Nelson et al., 2018). Verschueren and colleagues (2018) found that identity confusion positively predicted body dissatisfaction, whereas identity synthesis buffered against body dissatisfaction over time. In turn, body dissatisfaction positively predicted identity confusion and negatively predicted identity synthesis over time. Furthermore, a recent qualitative study revealed that the body can be salient in one’s identity in a negative and positive way (Kling, Wängqvist, & Frisén, 2018). People can identify with their body as an inseparable part of their identity. When this experience is positive, identity is described as feeling at home in one’s body (cf. Erikson, 1968).

This intricate link between identity formation and body image may be partially explained by body ideal internalization. Individuals who lack a clear sense of self, seek external sources to define themselves, and body ideals are very accessible sources for self-definition (Vartanian et al., 2018). Individuals with higher levels of identity disturbance are especially vulnerable to internalize body ideals (Vartanian et al., 2018). Verstuyf and colleagues (2014) also found that one’s identity style could render adolescents more or less vulnerable for adopting body ideals. An increase in body ideal internalization may result in body dissatisfaction, as these body ideals are very difficult to achieve (Dittmar, 2007; Stice, 2002).

### Body dissatisfaction as a predictor of ED symptomatology

A recent systematic review by Shagar and colleagues (2017) highlighted that body dissatisfaction is an important risk factor for the development of ED symptomatology in adolescents and emerging adults. Investigating precursors of ED symptomology is crucial, due to its high prevalence in adolescence and emerging adulthood. Research has indicated that 56–57% of adolescent girls and 28–31% of boys reported one or more weight-control behaviors (e.g., fasting, using laxatives, vomiting: Croll, Neumark-Sztainer, Story, & Ireland, 2002). Similarly, unhealthy weight-control behaviors (e.g., fasting, skipping meals, using a food substitute, cigarette smoking) have been reported by 57% of girls and 33% of boys, whereas extreme weight-control behaviors (e.g., using dieting pills, laxatives, vomiting) were reported by 12% of girls and 5% of boys (Neumark-Sztainer, Story, Hannan, Perry, & Irving, 2002). Furthermore, Quick and Byrd-Bredbenner (2013) found that 25% of emerging adults engaged in dietary restraint, 14% reported regular binge eating, and 33% mentioned inappropriate compensatory behaviors. In sum, research has demonstrated that body dissatisfaction plays an important role in ED symptoms, which are alarmingly high in adolescents and emerging adults.

Body dissatisfaction is also a major risk factor for clinical EDs. Body image disturbance constitutes a core diagnostic criterion for anorexia and bulimia nervosa (DSM-5; American Psychiatric Association [APA], 2013). Further, people who regard their body negatively might experience the body as an object separate from the self (Orbach & Mikulincer, 1998), which may give rise to self-destructive behaviors (Brausch & Muehlenkamp, 2007). These individuals may experience lower thresholds to harm their body to cope with distressing feelings (Brausch & Muehlenkamp, 2007; Muehlenkamp & Brausch, 2012). The more body dissatisfaction one experiences, the more likely that one develops ED symptoms as a way to regulate emotions.

In sum, these findings suggest that identity formation and ED symptomatology are significantly related to each other and that body dissatisfaction may constitute a mechanism through which identity formation predicts ED symptomatology.

## The Present Study

This cross-sectional study examined the mediating role of body dissatisfaction in the relationship between identity formation and ED symptomatology in mid-to-late adolescents and emerging adults. First, we hypothesized that identity disturbance and lack of identity would positively predict ED symptomatology, whereas identity consolidation would buffer against ED symptomatology (Verschueren, Claes, et al., 2018; Verschueren et al., 2017; Verstuyf et al., 2014). Second, we hypothesized that identity disturbance and lack of identity would positively predict body dissatisfaction, whereas identity consolidation would negatively predict body dissatisfaction (Kamps & Berman, 2011; Nelson et al., 2018; Vartanian et al., 2018; Verschueren, Claes, et al., 2018; Verstuyf et al., 2014; Wängqvist & Frisén, 2013). Third, we hypothesized that body dissatisfaction would positively predict ED symptomatology (Brausch & Muehlenkamp, 2014; Shagar et al., 2017; Stice, 2002). Combining our different expectations, we propose that the relationship between identity formation and ED symptomatology would be mediated by body dissatisfaction. We hypothesized that identity disturbance and lack of identity would positively predict body dissatisfaction, which in turn, would predict ED symptomatology. We do not formulate differential hypotheses for identity disturbance and lack of identity as no guiding research is available to formulate such differential hypotheses. We tentatively expect to find similar path coefficients, with possibly stronger path coefficients for lack of identity because this scale correlated highest with emotion dysregulation and psychopathology (Kaufman et al., 2015).

In addition, the present study also investigated whether the aforementioned mediation model differs across gender (males and females) and age groups (mid-to-late adolescents and emerging adults). First, compared to males, females generally experience more body dissatisfaction, ED symptomatology (Cash & Smolak, 2011), and identity confusion (Verschueren, Rassart, Claes, Moons, & Luyckx, 2018). However, recent evidence revealed that bidirectional effects between identity formation on the one hand and body dissatisfaction and ED symptomatology on the other hand were not significantly different in males and females (Verschueren, Claes, et al., 2018). Although these first results are promising, there is still a need to replicate these results in future research. Therefore, we do not formulate strong hypotheses in this respect. Second, the present study samples mid-to-late adolescents (15–18 years) and emerging adults (18–30 years). An ongoing identity search has been more strongly associated with depressive symptoms and identity distress with increasing age, with the strongest associations occurring in the late 20s as opposed to adolescence and the early twenties (Luyckx, Klimstra, Duriez, Van Petegem, & Beyers, 2013; Palmeroni et al., 2019). Accordingly, we expect to find stronger directional paths from identity disturbance and lack of identity to body dissatisfaction and ED symptomatology in emerging adults as identity problems are assumed to result in higher levels of distress and symptomatology in older individuals (as compared to mid-to-late adolescents).

## Methods

### Participants and Procedure

We combined two samples which were collected in 2017 in Flanders (the Dutch-speaking part of Belgium). The total sample size comprised 659 participants (68.9% females; M_age_ = 19.44; SD_age_ = 3.99). The first sample consisted of 327 adolescents aged 15–18 years (71.38% females; M_age_ = 15.99; SD_age_ = 0.96) who filled out self-report questionnaires during school hours in one high school. All participants under the age of 18 years received parental consent and provided informed assent themselves. Participants above the age of 18 provided informed consent themselves. The second sample consisted of 332 emerging adults aged 18–30 years (69% females; M_age_ = 22.82; SD_age_ = 2.72) who filled out the questionnaires via an online survey (*Limesurvey*). All individuals participated voluntarily, signed an informed consent form, and anonymity was guaranteed in this study approved by the Social and Societal Ethics Committee of KU Leuven (reference numbers of approval: G- 2016 09 632, G- 2016 09 626).

### Measures

#### Identity

To assess identity along a continuum ranging from healthy identity functioning to clinical identity disturbance, the 27-item Dutch version of the Self-concept and Identity Measure (SCIM) was used (Kaufman et al., 2015). The SCIM consists of three subscales: identity consolidation, identity disturbance, and lack of identity. Identity consolidation refers to healthy identity development in which individuals feel certain about who they are and experience themselves as an integrated whole, similar to the notion of identity synthesis (Erikson, 1968). Identity disturbance assesses identity-related issues such as feelings of discontinuity and confusion, whereas lack of identity refers to pathological identity disturbance including individuals who feel broken and empty inside (Kaufman et al., 2015). The SCIM has been validated in adults from the US and Belgium (Bogaerts et al., 2018; Kaufman et al., 2015). All items were scored on a seven-point Likert scale ranging from ‘1’ (*strongly disagree*) to ‘7’ (*strongly agree*). Sample items include: *“I know what I believe or value”* (identity consolidation), *“I imitate other people instead of being myself”* (identity disturbance), and *“I feel empty inside, like a person without a soul”* (lack of identity). Cronbach’s alpha coefficients were .70 for identity consolidation, .79 for identity disturbance, and .90 for lack of identity.

#### Body dissatisfaction

Body image was evaluated using the body feelings and attitudes subscale of the *Body Investment Scale* (BIS; Orbach & Mikulincer, 1998) and the body dissatisfaction subscale of the *Eating Disorder Inventory-3* (EDI-3; Garner, 2004). The BIS is validated in US community adolescents (Osman et al., 2010). The original English version of the questionnaire was translated into Dutch by using the translation/back-translation procedure. The body feelings and attitudes scale of the BIS measures feelings that individuals experience regarding their own body and is closely related to body (dis)satisfaction (Orbach & Mikulincer, 1998). The scale consists of six items to be rated on a five-point Likert scale ranging from ‘1’ (*I do not agree at all*) to ‘5’ (*Strongly agree*). Sample items include: *“I am satisfied with my appearance”*. Cronbach’s alpha coefficient was .92. The body dissatisfaction subscale of the EDI-3 measures the degree to which an individual is convinced that specific body parts (e.g., hips, thighs) are too large and is unsatisfied with his/her shape. Sample items include: *“I think that my stomach is too big”*. The scale consists of 9 items to be rated on a six-point Likert scale ranging from ‘1’ (*Never*) to ‘6’ (*Always*). Cronbach’s alpha coefficient was .93. A significant positive correlation was found between the two subscales [*r*(625) = .72, *p* < .001].

#### ED symptomatology

The *Eating Disorder Inventory-3* (EDI-3; Garner, 2004) is a valid questionnaire to tap into both body dissatisfaction (as mentioned above) and various other ED symptoms (Lehmann et al., 2013; Nyman-Carlsson, Engström, Norring, & Nevonen, 2015). The drive for thinness and bulimia scales of the EDI-3 Risk Scales were used. Drive for thinness indicates a strong desire to have a thin body with a low amount of fat, resulting in an overestimation of one’s own body weight and size (APA, 2013). Bulimia measures the presence of binge eating episodes (i.e., the consumption of a large amount of food during a limited period of time accompanied by a lack of control) and compensatory behaviors to prevent weight gain (e.g., purging and vomiting; APA, 2013). Both subscales consist of 7 items which were scored on a six-point Likert scale ranging from ‘1’(*never*) to ‘6’(*always*). Sample items include: *“I feel extremely guilty after overeating”* (drive for thinness) and *“I eat when I am upset”* (bulimia). Cronbach’s alpha coefficients were .91 for drive for thinness and .80 for bulimia.

#### Body Mass Index (BMI)

All participants reported their weight and height and BMI was calculated using the following formula (weight in kilogram/height*height in meters).

### Primary Statistical Analyses

Our primary hypotheses were investigated using structural equation modeling within a latent variable framework in MPLUS version 8.1 (Muthén & Muthén, 1998–2012). All models were estimated using robust maximum likelihood estimation (MLR) to account for non-normality (Kline, 2015). Following fit indices were used (Browne & Cudeck, 1993; Hu & Bentler, 1999; Kline, 2015): Yuan-Bentler scaled χ^2^, which should be as small as possible; a normed Yuan-Bentler scaled χ^2^ divided by its degrees of freedom was also calculated, which should be equal or less than 3; Root Mean Square Error of Approximation (RMSEA), which should be less than .08; Standardized Root Mean Square Residual (SRMR), which should be less than .09; and Comparative Fit Index (CFI), which should exceed .90 for adequate fit.

In order to investigate our primary hypotheses within a latent variable framework, we constructed a measurement model which represents manifest variables (questionnaire items) as indicators of underlying factors. In order to reduce model complexity and the number of indicators for each latent variable to the optimal number of three (Little, Cunningham, Shahar, & Widaman, 2002), parcels consisting of multiple items were used as indicators. Furthermore, item parcels scores, in general, are more normally distributed than individual item scores (Little et al., 2002). In creating these parcels, we used the item-to-construct balance parceling method (Little et al., 2002). For identity consolidation, two parcels consisted of three items and one parcel of four items; for identity disturbance, two parcels consisted of four items and one parcel of three items; and for lack of identity, all three parcels consisted of two items. For body dissatisfaction, the three parcels consisted of five items. Finally, both for drive for thinness and bulimia, two parcels consisted of two items and one parcel of three items.

For the structural part of the model, three primary models were estimated (Holmbeck, 1997): (a) a direct effects model including identity formation as a predictor of drive for thinness and bulimia; (b) a full mediation model in which identity formation is indirectly related to these outcomes through body dissatisfaction; and (c) a partial mediation model including both direct paths from identity formation to outcomes that were significant in the direct effects model, and indirect paths through body dissatisfaction. In all models, apart from these directional paths, all associations among the three identity formation scales and between drive for thinness and bulimia were included. Gender and age were controlled for in all models by regressing all latent variables on gender and age. In an auxiliary analysis, BMI was additionally controlled for. To increase model parsimony, only significant paths from the control variables to the study variables were retained. The Model Indirect command was used to examine the significance of indirect effects.

To examine gender and age differences in the primary models, multi-group analyses were conducted. We investigated if the measurement and structural model (i.e., factor loadings and path coefficients) could be set equal across males/females and across mid-to-late adolescents (15–18 years) and emerging adults (18–30 years). We compared the fit of the fixed model with constrained coefficients (coefficients were constrained as equal across groups) to the fit of the free model with unconstrained coefficients (coefficients could be different across groups) by checking differences in comparative fit indices. A significantly better fit of the free model in comparison with the fixed model can be concluded when at least two of the following criteria were satisfied: a significant Yuan-Bentler scaled Δχ^2^ (*p* < 0.05), ΔRMSEA ≥ 0.015, and ΔCFI ≥ .01.

## Results

### Preliminary Analyses

Preliminary analyses were conducted in SPSS version 26. Multivariate analyses of variance (MANOVA) indicated gender differences [*F*(6,593) = 25.614, *p* = .000, partial η^2^ = .199] and differences between age groups (mid-to-late adolescents and emerging adults) [*F*(6,593) = 6.809, *p* = .000, partial η^2^ = .064] in the study variables (based on Wilks’ Lambda). First, follow-up univariate analyses showed that boys scored higher on identity consolidation, whereas girls scored higher on lack of identity, body dissatisfaction, drive for thinness and bulimia (Table 1). Second, follow-up univariate analyses indicated that emerging adults scored higher on identity consolidation and lack of identity, whereas mid-to-late adolescents scored higher on identity disturbance (Table 1). Table 2 presents Pearson correlations among all study variables. Identity consolidation was negatively related to body dissatisfaction, drive for thinness and bulimia. Both identity disturbance and lack of identity were positively related to body dissatisfaction, drive for thinness, and bulimia.

**Table 1:** Descriptive Statistics and Mean-Level Differences based on Analysis of Variance.

	Total Sample	Gender Differences			Age Group Differences				

		Males	Females		Partial	Mid-to-late adolescents	Emerging adults		Partial

	*M*(*SD*)	*M*(*SD*)	*M*(*SD*)	*F* Ratio	η^2^	*M*(*SD*)	*M*(*SD*)	*F*Ratio	η^2^

Identity consolidation	5.12 (0.74)	5.22 (0.79)	5.08 (0.71)	4.78*	.008	5.03 (0.86)	5.20 (0.71)	4.01*	.007
Identity disturbance	2.99 (0.89)	2.95 (0.93)	3.01 (0.87)	0.64	.001	3.17 (0.83)	2.84 (0.90)	7.98**	.013
Lack of identity	2.35 (1.26)	2.19 (1.20)	2.43 (1.26)	4.85*	.008	2.31 (1.24)	2.40 (1.30)	4.59*	.008
Body dissatisfaction	3.31 (1.25)	2.48 (1.02)	3.68 (1.16)	161.13***	.198	2.96 (1.10)	2.96 (0.96)	1.38	.002
Drive for thinness	2.69 (1.21)	1.96 (0.96)	3.01 (1.17)	125.15***	.160	2.68 (1.32)	2.72 (1.10)	3.62	.006
Bulimia	2.12 (0.78)	1.89 (0.71)	2.22 (0.79)	26.44***	.039	2.17 (0.80)	2.05 (0.75)	1.32	.002

*Note*: M = mean; SD = standard deviation; F = F-value; Partial η^2^ = partial eta squared. * *p* < .05. ** *p* < .01. ***p < .001.

**Table 2:** Pearson Correlations between Study Variables.

	Identity consolidation	Identity disturbance	Lack of identity	Body dissatisfaction	Drive for thinness	Bulimia

Identity consolidation	1					
Identity disturbance	–.60***	1				
Lack of identity	–.65***	.63***	1			
Body dissatisfaction	–.35***	.29***	.47***	1		
Drive for thinness	–.21***	.27***	.34***	.82***	1	
Bulimia	–.30***	.44***	.37***	.51***	.60***	1

*** *p* < .001.

### Primary Analyses: Direct Effects Models

For the path analyses, the measurement model provided an adequate fit [χ^2^(80) = 234.12, *p* < .001; χ^2^/df = 2.93; CFI = .962; RMSEA = .054; SRMR = .039]. Standardized factor loadings for the different parcels ranged from .51 to .91 (all *p*s < .001).

**Figure 1:**
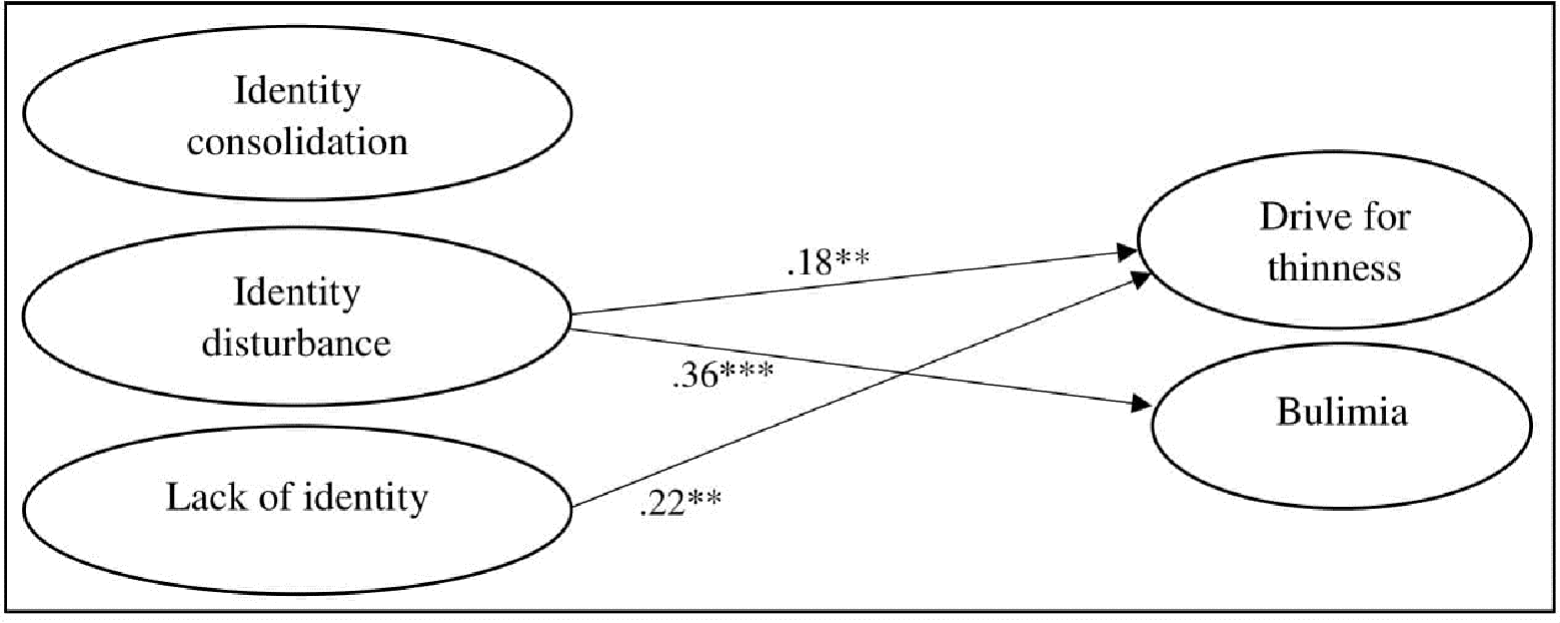
Direct effects model including all significant directional paths among the study variables. ** *p* < .01. *** *p* < .001.

With respect to the final model including both the measurement and structural part (with non-significant paths from age and gender trimmed), adequate fit was obtained (χ^2^(103) = 346.094, *p* < .001; χ^2^/df = 3.36; CFI = .946; RMSEA = .060; SRMR = .043). In this model, gender (dummy coded with 0 = boys, 1 = girls) positively predicted lack of identity (β = .09, *p* < .01), drive for thinness (β = .40, *p* < .001), and bulimia (β = .17, *p* < .001). Gender negatively predicted identity consolidation (β = –.11, *p* < .05). Age positively predicted identity consolidation (β = .23, *p* < .001) and drive for thinness (β = .10, *p* < .01). All significant directional paths among the study variables are displayed in Figure 1. Identity disturbance positively predicted drive for thinness (β = .18, *p* < .01) and bulimia (β = .36, *p* < .001), whereas lack of identity positively predicted drive for thinness (β = .22, *p* < .01).

Multi-group analyses for gender and age indicated that the coefficients could be set equal for males and females [∆Yuan-Bentler scaled-χ^2^(6) = 2.19, *p* = 0.902; ΔCFI = .002; ΔRMSEA = .002], as well as for mid-to-late adolescents and emerging adults [∆Yuan-Bentler scaled-χ^2^(6) = 16.55, *p* = 0.011; ΔCFI = .002; ΔRMSEA = .001].

Auxiliary analysis including BMI as an additional control variable [with only significant paths from BMI to the study variables being retained in the model; χ^2^(114) = 366.33, *p* < .001; χ^2^/df = 3.21; CFI = .945; RMSEA = .059; SRMR = .043] resulted in virtually identical findings as the model displayed in Figure 1, with all paths displayed remaining significant. Additionally, BMI positively predicted lack of identity (β = .08, *p* < .05), drive for thinness (β = .42, *p* < .001), and bulimia (β = .35, *p* < .001).

### Primary Analyses: Mediation Models

**Figure 2:**
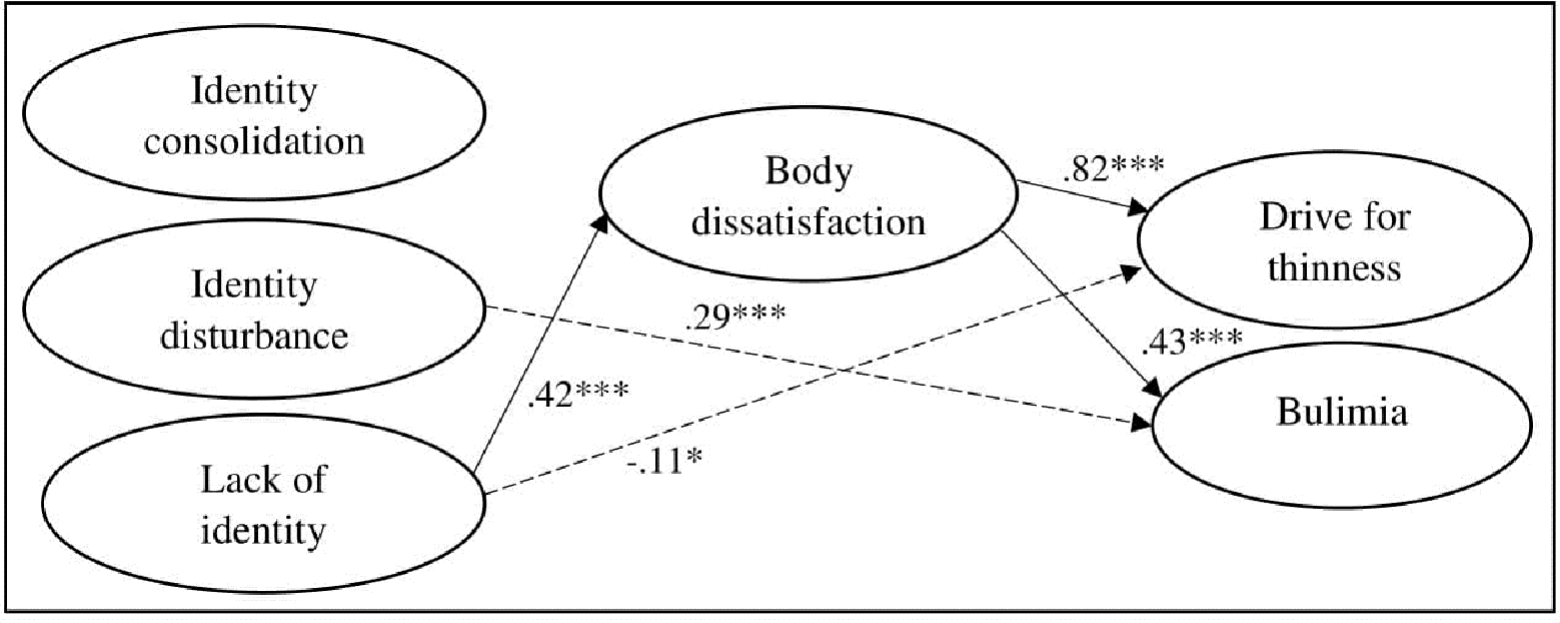
Partial mediation model including all significant directional paths among the study variables. * *p* < .05. *** *p* < .001.

The measurement model provided an adequate fit to the data [χ^2^(120) = 311.409, *p* < .001; χ^2^/df = 2.60; CFI = .970; RMSEA = .049; SRMR = .038]. Standardized factor loadings for the different parcels ranged from .51 to .97 (all *p*s < .001). The final full mediation model including both the measurement and structural part (with non-significant paths from age and gender trimmed) provided an adequate fit to the data [χ^2^(155) = 492.584, *p* < .001; χ^2^/df = 3.18; CFI = .951; RMSEA = .058; SRMR = .057]. Next, when including the original significant direct paths, adequate fit was obtained for the final model (χ^2^(152) = 448.060, *p* < .001; χ^2^/df = 2.95; CFI = .957; RMSEA = .055; SRMR = .044). Gender (dummy coded with 0 = boys, 1 = girls) positively predicted lack of identity (β = .09, *p* < .01), body dissatisfaction (β = .38, p < .001), and drive for thinness (β = .08, *p* < .01), and negatively predicted identity consolidation (β = –.11, *p* < .05). Age positively predicted identity consolidation (β = .24, *p* < .001), and negatively identity disturbance (β = –.24, *p*<.001) and bulimia (β = –.08, *p* < .05). All significant directional paths among the study variables are displayed in Figure 2.

Identity disturbance positively predicted bulimia (β = .29, *p* < .001) and lack of identity negatively predicted drive for thinness (β = –.11, *p* < .05). Lack of identity positively predicted body dissatisfaction (β = .42, *p* < .001). Body dissatisfaction, in turn, positively predicted drive for thinness (β = .82, *p* < .001) and bulimia (β = .43, *p* < .001). Indirect effects linking lack of identity to drive for thinness and bulimia via body dissatisfaction were significant at *p* < .001.

Multi-group analyses for gender and age indicated that the coefficients could be set as equal for males and females [∆Yuan-Bentler scaled-χ^2^(8) = 6.26, *p* = 0.618; ΔCFI = .001; ΔRMSEA = .001], as well as for mid-to-late adolescents and emerging adults [∆Yuan-Bentler scaled-χ^2^(8) = 21.95, *p* = 0.005; ΔCFI = .001; ΔRMSEA = .002].

Auxiliary analysis including BMI as an additional control variable [with only significant paths from BMI to the study variables being retained in the model; Yuan-Bentler scaled-χ^2^(165) = 472.110, *p* < .001; χ^2^/df = 2.86; CFI = .956; RMSEA = .054; SRMR = .043] resulted in virtually identical findings as the model displayed in Figure 1, with all paths displayed remaining significant. Additionally, BMI positively predicted lack of identity (β = .13, *p* < .05), drive for thinness (β = .27, *p* < .001), and bulimia (β = .19, *p* < .01), and negatively predicted body dissatisfaction (β = –.24, *p* < .001). Furthermore, drive for thinness was positively predicted by identity disturbance (β = .11, *p* < .05) and lack of identity (β = –.11, *p* < .05).

## Discussion

The present study examined the mediational role of body dissatisfaction between identity formation and ED symptomatology in mid-to-late adolescents and emerging adults. The present findings indicated that identity formation significantly predicted ED symptomatology directly. Furthermore, indirect effects were also found linking identity formation to ED symptomatology through the mechanism of body dissatisfaction. More specifically, lack of identity predicted both drive for thinness and bulimia through the intervening mechanism of body dissatisfaction. Additional multigroup analyses revealed no significant differences on direct or indirect effects between males and females or between mid-to-late adolescents and emerging adults.

### Identity Formation as a Predictor for ED symptomatology

As expected, identity disturbance positively predicted drive for thinness and bulimia, whereas lack of identity only positively predicted drive for thinness. This identity-ED symptomatology pathway did not differ across gender and age groups. These findings are partially in line with Verschueren and colleagues (2018) who found that identity disturbance was a significant predictor for bulimia, but not for drive for thinness in adolescents. The inconsistent results regarding identity disturbance and drive for thinness might be explained by the ambiguous nature of drive for thinness in identity functioning (Bruch, 1981; Casper, 1983; Verschueren, Claes, et al., 2018). It has been suggested that drive for thinness could be possibly related to both identity synthesis and identity disturbance (Verschueren, Claes, et al., 2018). Striving for thinness can become a key identity goal that can provide a stronger sense of identity, while an overvaluation of thinness and eating regulation in identity can also result in a more vulnerable sense of identity (Corning & Heibel, 2016; Verschueren et al., 2019). Hence, this rather complicated relationship between drive for thinness and identity might help understanding the conflicting findings in empirical research.

These findings fit well with theories on ED symptomatology development and the role of identity formation. First, bulimia (i.e., binge eating and purging behaviors) have been put forward as maladaptive coping behaviors to regulate identity-related feelings of stress and uncertainty (Wheeler et al., 2001). This theorizing concurs with previous findings indicating that identity problems are associated with emotion dysregulation (Kaufman et al., 2014). Second, concerning drive for thinness, it has been stated that the pursuit of thinness can become a key identity goal in individuals, leading to behaviors such as controlling food intake and body weight. Especially individuals who struggle with their own identity process may strive for thinness as a source of self-definition (Schupark-Neuberg & Nemeroff, 1993), as thinness is portrayed as key towards happiness in Western societies (Dittmar, 2007).

### Body Dissatisfaction as a Mediator between Identity Formation and ED symptomatology

Apart from direct effects linking identity formation to ED symptomatology, the present study indicated that body dissatisfaction may play a mediating role during mid-to-late adolescence as well as during emerging adulthood. Statistically significant indirect effects were found linking identity formation to ED symptomatology through the intervening mechanism of body dissatisfaction.

First, lack of identity was indirectly related to drive for thinness and bulimia via body dissatisfaction. Research has indicated that especially individuals who lack a clear identity are more susceptible to turn to external standards, such as appearance ideals, to derive a sense of identity (Stice, 1994; Verstuyf et al., 2014). In this regard, one’s identity then becomes reduced to the external appearance of one’s own body, which almost invariably leads to feelings of body dissatisfaction (Dittmar, 2007). Furthermore, body dissatisfaction positively predicted both drive for thinness and bulimia, in line with previous findings (Muehlenkamp, Peat, Claes, & Smits, 2012; Stice, 1994; Striegel-Moore, Silberstein, & Rodin, 1986).

Another possible explanation is that body image investment, in addition to body dissatisfaction, plays an important role in this relationship. Individuals lacking an identity might counteract the inner emptiness they experience by investing more in appearance as a primary source of self-worth (Corning & Heibel, 2016; Stice, 1994; Vartanian et al., 2018). Body image investment refers to the importance of the body for self-evaluation. We propose that further research should investigate the possible role of body image investment as an additional moderator in the current mediation model. More specifically, the mediating role of body dissatisfaction between identity formation and ED symptomatology may be conditional on one’s level of body image investment. It could be possible that lack of identity is significantly related to body dissatisfaction, especially in individuals scoring high on body image investment.

Second, contrary to our hypothesis, no mediating effect of body dissatisfaction was found for identity disturbance. However, the direct effect from identity disturbance to bulimia remained significant in the partial mediation model. These results suggest that this relationship might be explained by other mediators, such as emotion dysregulation. Previous findings indicated that identity problems and emotion dysregulation are associated (Kaufman et al., 2014). ED symptomatology, such as binging, purging, and restrictive eating patterns have been forwarded as ways to cope with such negative emotions, as they provide ways to escape, avoid or distract from these emotions (Schupark-Neuberg & Nemeroff, 1993; Wheeler et al., 2001).

Third, the analyses did not show any significant direct or indirect pathways from identity consolidation to body dissatisfaction or ED symptomatology. Although no significant association occurred between identity consolidation and body dissatisfaction, identity consolidation may affect other components of body image, such as positive body image. Positive body image entails favorable opinions toward one’s body, body acceptance, competence and respect toward the body and does not represent the absence of body dissatisfaction (Tylka & Wood-Barcalow, 2015). Research has indicated that individuals with a strong personal identity appear to have stronger self-esteem (Luyckx et al., 2013), which is positively associated with positive body image (Tylka & Wood-Barcalow, 2015). Hence, future research should assess body image as a multidimensional construct in studying relations with identity formation.

Lastly, the present study found no significant differences between males and females or between mid-to-late adolescents and emerging adults on the pathway from identity formation to ED symptomatology through body dissatisfaction. The presents findings point to the fact that although females score higher on lack of identity, body dissatisfaction and ED symptomatology, both males and females who experience identity problems may be at risk to develop body dissatisfaction and/or ED symptomatology. These results emphasize how important it is to include boys/males in research on body dissatisfaction and ED symptomatology as well. Up till now, males are often underrepresented in research on body dissatisfaction and ED symptomatology. Furthermore, the present findings support the view that body dissatisfaction plays a significant role in the relationship between identity formation and ED symptomatology in both mid-to-late adolescents and emerging adults. This concurs with previous findings on the importance of body dissatisfaction in these life periods. Adolescence and emerging adulthood are characterized by extensive bodily changes (Cash & Smolak, 2011), resulting in a heightened focus on the body (Arnett, 2000). During this life phase, the body can become a purposeful pathway through which distressing emotions can be expressed. This is in line with our results indicating that people experiencing lack of identity, may express feelings of emptiness and insecurity through their own body, by, for instance, focusing on body ideals. This heightened focus on the body, or ED symptomatology, can be regarded as means to avoid dealing with profound identity work for both males and females experiencing issues regarding identity formation.

### Implications

#### Theoretical implications

While most theories and studies focus on either the role of identity or body dissatisfaction in ED symptomatology development, current research demonstrated an interplay between identity and body dissatisfaction in the prediction of ED symptomatology. Although our results are promising, future studies are recommended in order to verify the interrelations between identity formation, body dissatisfaction, and ED symptomatology from a longitudinal perspective.

#### Practical implications

There is a clear lack of emphasis on building and strengthening a positive, self-endorsed identity in prevention and intervention programs targeting ED symptomatology (Corning & Heibel, 2016). Provided that the present findings are replicated longitudinally, they demonstrate that identity formation, together with body dissatisfaction, should be taken into account in such programs. First, the promotion of a positive body image may be one avenue to prevent body dissatisfaction and ED symptomatology by increasing favorable opinions toward one’s body (Tylka & Wood-Barcalow, 2015). The current findings indeed indicated that body dissatisfaction may play a significant role in the relationship between identity formation and ED symptomatology in mid-to-late adolescents and emerging adults. However, when positive body image is promoted, the attention is again shifted towards the body as a necessary source of self-esteem. Therefore, attention must be paid to also focus on aspects of the self that are unrelated to body/appearance.

Second, strengthening one’s identity may prevent or reduce ED symptomatology through a decrease in body dissatisfaction. Hence, it might be useful to identify individuals experiencing lack of identity, as these individuals might be especially vulnerable to turn to appearance ideals in an effort to build their identity. When identity is narrowly based on one aspect of the self, such as appearance, threats to that aspect also threaten the identity more broadly because other sources of self-esteem are lacking. It is important that young people’s identities become less closely linked to appearance by enhancing other identity aspects (as proposed by Corning and Heibel (2016)). This way, individuals can rely on a more diversified sense of self which is shifted away from body and appearance.

### Limitations

First, due to the cross-sectional design, no authoritative claims regarding development or directionality of effects can be made. ED symptomatology may also lead to more body dissatisfaction and identity problems. Although scarce, previous research already revealed a reciprocal relationship between identity formation and ED symptomatology (Verschueren, Claes, et al., 2018). To reach more definite conclusions, a longitudinal study is warranted in which individuals are followed from early adolescence until their late twenties.

Second, we only used self-report questionnaires. Although the use of self-report questionnaires is preferable to assess internal/behavioral constructs, the inclusion of alternative data-collection methods could provide additional information (e.g., interview, significant others reports).

Third, in this study we did not capture the full spectrum of bodily concerns and ED symptomatology in males and females. The focus of our study was mainly on bodily concerns and ED symptomatology which are common in clinical EDs anorexia nervosa and bulimia (as measured by the EDI-3). These bodily concerns and ED symptomatology are encountered more by females than males as women generally experience a higher ‘drive for thinness’, whereas men are especially vulnerable for ‘a drive for muscularity and leanness’. Although the EDI-3 successfully assesses leanness (and low body fat), we did not account for bodily concerns regarding muscularity or eating behaviors aimed at achieving the muscular ideal in this study. Hence, we expect an underestimation of bodily concerns and ED symptomatology in males in our study. Future studies should include additional measurements that can assess the full spectrum of bodily concerns and ED symptomatology in both males and females, such as drive for muscularity, muscularity dissatisfaction, compulsive exercise, the use of protein supplements, and anabolic steroids.

Fourth, regarding body image, our study focused primarily on evaluative body image measurement by assessing body dissatisfaction. However, research would benefit from assessing body image as a broader construct by measuring additional body image dimensions such as positive body image, embodiment, and body image investment.

## Conclusion

The present cross-sectional study indicated that body dissatisfaction plays an important role in the relationship between identity formation and ED symptomatology during mid-to-late adolescence and emerging adulthood. More specifically, lack of identity seems especially important in the prediction of ED symptomatology through the intervening mechanism of body dissatisfaction. Multigroup analyses revealed no significant differences between males and females or between adolescents and emerging adults on direct or indirect effects. The results of the present study are promising and should be validated in future research using a longitudinal design.

## Data Accessibility Statement

Data used in the present project are not publicly available due to privacy or ethical restrictions. The data are available on (motivated) request from the corresponding author.
